# Training Load in Different Age Category Soccer Players and Relationship to Different Pitch Size Small-Sided Games

**DOI:** 10.3390/s21155220

**Published:** 2021-07-31

**Authors:** Fernando J. Santos, Teresa P. Figueiredo, Dalton M. Pessôa Filho, Carlos E. L. Verardi, Anderson G. Macedo, Cátia C. Ferreira, Mário C. Espada

**Affiliations:** 1 Faculty of Human Kinetics, University of Lisbon, 1499-002 Cruz Quebrada, Portugal; fernando.santos@ese.ips.pt; 2 Polytechnic Institute of Setúbal, School of Education, 2914–504 Setúbal, Portugal; teresa.figueiredo@ese.ips.pt (T.P.F.); catia.ferreira@ese.ips.pt (C.C.F.); 3 Life Quality Research Centre, Complexo Andaluz, Apartado, 2040-413 Rio Maior, Portugal; 4 Department of Physical Education, São Paulo State University (UNESP), Bauru 17033-360, Brazil; dalton.pessoa-filho@unesp.br (D.M.P.F.); carlos.verardi@unesp.br (C.E.L.V.); andersongmacedo@yahoo.com.br (A.G.M.); 5 Graduate Programme in Human Development and Technology, São Paulo State University (UNESP), Rio Claro 13506-900, Brazil; 6 Graduate Programme in Developmental Psychology and Learning, Faculty of Science, São Paulo State University (UNESP), Bauru 17033-360, Brazil; 7 Research Group in Optimization of Training and Sport Performance (GOERD), Faculty of Sports Sciences, University of Extremadura, 10003 Caceres, Spain

**Keywords:** soccer, age categories, small-sided games, running speed, metabolic power

## Abstract

This study sought to evaluate the training load in different age category soccer players associated with distinct pitch size small-sided games (SSGs). Twenty-four soccer players (eight in each age category: U-12, U-15, and U-23) performed three consecutive 4 vs. 4 ball possession SSGs (SSG1: 16 × 24 m; SSG2: 20 × 30 m; and SSG3: 24 × 36 m) all with 3 min duration and 3 min rest. Subjects carried ultra-wideband-based position-tracking system devices (WIMU PRO, RealTrack System). Total distance covered increased from SSG1 to SSG3 in all age categories and predominantly in running speeds below 12 km·h^−1^. Moreover, distance covered in 12–18 km·h^−1^ running speed was different in all performed SSGs and age categories. Residual or null values were observed at 18–21 km·h^−1^ or above running speed, namely in U-12, the only age category where metabolic power and high metabolic load distance differences occurred throughout the performed SSGs. Edwards’ TRIMP differences between age categories was only observed in SSG2 (U-12 < U-15). The design of SSGs must consider that the training load of the players differs according to their age category and metabolic assessment should be considered in parallel to external load evaluation in SSGs. Wearable technology represents a fundamental support in soccer.

## 1. Introduction

Monitoring of training demands is crucial to optimize daily tasks and competitive performance in sport. Soccer has become worldwide popular, resulting in an increase in the number of season competitions and games involving highly prepared and specialized players. Specific physical fitness attributes of the athletes—including agility, speed, and strength—deserve specific approaches and detailed analysis in scientific research [1]. Soccer is a team sport that requires an efficient collective organization and, simultaneously, specific development of each player, with consideration of individual and group perspectives [2], since soccer is associated with complexity in collective and individual actions performed by the players [3]. Consequently, the accurate assessment of an individual’s training load represents an essential component of effective training prescription since the training process in team sports must provide stimuli to a wide range of physical, tactical, and technical components [4]. Recent studies attempted to highlight the positive impact of small-sided games (SSGs) in stimulating such components [5]. In soccer, coaches manipulate training constraints on a daily basis, whether in the lab (e.g., isokinetic assessments), in the gym (e.g., rehabilitation), or on the pitch (e.g., SSGs) [6]. These smaller game formats are associated with more specificity in soccer, and fortunately wearable technology has progressed and its presence in sports has increased—specifically, through the use of global positioning systems (GPS) during daily training. This current possibility has provided the opportunity to derive valid and reliable estimates of several relevant soccer task-related variables, including the speed attained and the distance covered during a range of activities [7].

The proper selection of the SSG for training in grassroots sport will allow better adaptation of the training to the demands of the match [8], thus reducing the risk of injury and improving the physical condition of the players—an essential element in their correct development and learning [9]. Such multidimensional effects of SSGs on players’ acute responses make these games highly popular and frequently used by coaches for players of different age categories, experience, and competitive levels [10,11]. By changing task constraints during SSGs, coaches may directly amplify or inhibit the range of players’ action possibilities within the context of practice [12], where manipulation of the playing area represents the variable which most influence players’ external workload and technical performance, and consequently, decision making [13]. For this reason, how the body responds to the physical demands of the game—e.g., sprinting, running at different intensities—are of great interest for the literature [14].

Limitations associated with SSGs were indicated in producing high-speed activities [15] because they are played on smaller pitch areas [16,17] where the players do not have enough space to reach their maximal sprinting speed. More recently, it was observed that in overall terms, SSGs typically present lower demands relating to high-speed running (HSR) and sprint actions than competitive matches of professional soccer players do [18]. Nevertheless, HSR and sprint should form an integral part of the player monitoring process [19]. Additionally, the metabolic power (*P*_met_) approach has been employed to examine the physical demands of training sessions [20], SSGs [21], and match play [22] in elite professional soccer players. This approach involves accelerations and decelerations, whereas the traditional speed approach only includes distances covered at constant speeds [23] and is strongly related to energy expenditure [24].

To the best of our knowledge, no study has adopted the approach of quantifying the metabolic and mechanical demands of different SSGs in different age category soccer players during the performance of distinct pitch size SSGs. Traditionally, U-12 and U-15 soccer players have different weekly training conditions (number of days and session duration) and even competitive conditions (game time and number of players per team), facts that also transfer to the U-23 age category, where a significant number of soccer players are professionals—a status that is reflected in their daily training. It is unquestionably relevant to provide safe and appropriate training structures to enhance the performance of players in different age categories, consistent with the specific physical requirements of competitive match-play. Hence, the aim of this study was to evaluate the training load in different age category soccer players and its association with distinct pitch size SSGs.

## 2. Materials and Methods

### 2.1. Study Design and Setting

Players from different age categories (U-12, U-15, and U-23) were selected and evaluated in a single session to ensure the same environmental conditions (temperature, humidity), near the end of the 2018–2019 season (June 2019). The tasks were performed on natural grass floor (in very good condition) and games occurred in the afternoon period, with an average temperature of 22 °C and relative humidity of 60%. Twenty-four hours prior to the experimental session, the players were instructed to maintain their usual habits, which included eight hours of sleep the night before the data collection session and maintaining their nutritional routine. Players could drink fluids throughout the recovery time periods in the SSGs.

### 2.2. Participants

A total of twenty-four players were involved in the study. The sample size was calculated prior to the study for an alpha of 0.05 and a power of 0.8; the estimated sample was eighteen. Players were recruited as follows: sixteen players (U-12, n = 8, and U-15, n = 8) from the same club and the remaining eight U-23 soccer players from another club, in both cases national- or international-level clubs and prominent in soccer player development from age categories to senior level in Portugal.

The inclusion criteria for the players participating in the SSGs were: (1) players without injuries in the last two months; (2) players had participated in all training sessions in the six weeks prior to data collection; and (3) players had participated in the total playing time in the month of competition prior to data collection. The U-23 (age: 20.1 ± 1.5 years old; height: 1.83 ± 0.04 m; total body mass: 76.1 ± 3.6 kg; body fat: 10.1 ± 2.2%; and soccer experience: 13.3 ± 1.5 years), U-15 (age: 14.7 ± 0.8 years old; height: 1.68 ± 0.05 m; total body mass: 57.3 ± 3.9 kg; body fat: 10.5 ± 2.3%; and experience: 7.6 ± 1.3 years), and U-12 (age: 11.69 ± 0.5 years old; height: 1.59 ± 0.09 m; total body mass: 38.76 ± 4.21 kg; body fat: 11.3 ± 2.5%; and experience: 5.2 ± 1.9 years) groups were previously instructed on the tasks to be performed in the training sessions and were familiar with SSGs from throughout their training sessions and soccer playing careers. On average, U-23 players practiced 10–11 months per year with 5–6 weekly training sessions and played one match per week at the time of the data collection, but in some specific periods of the competitive season they played two matches per week. U-15 and U-12 trained four times a week plus one match every weekend. U-15 soccer players’ training sessions were 90 min and U-12 players’ sessions were 60 min. The official games of U-15 were characterized by 11-a-side games of two 35-minute periods, and those of U-12 were 7-a-side games with 30 min in each of the two playing periods.

Our study was conducted in accordance with the international ethical standards for sport and exercise science research [25] and in accordance with the Declaration of Helsinki. This study was submitted to the Ethical Committee of São Paulo State University (UNESP), which was registered and approved under (CAAE: 02523412.4.0000.5398, final report no: 237.707). Informed consent was obtained from all subjects, particularly in the case of U-15 and U-12 soccer players participating in the study, where consent was obtained from the parents/guardians.

### 2.3. Procedures

Training sessions started with a 25-min standardized warm-up, consisting of 5 min of slow jogging and strolling locomotion followed by 12 min of specific soccer drills, finishing with 3 min of progressive sprints and accelerations. Agility and speed drills were also conducted and a 5-minute ball possession game within a space of 20 × 20 m concluded the warm-up.

For data collection, players carried Wimu Pro ultra-wideband-based position-tracking system devices (RealtrackSystems, Almería, Spain). The system provides high ecological relevance for analysis of real training and match situations related to physical demands [26], and was previously validated in other studies on soccer [27] and basketball [28] where it was indicated as a valid and reliable inertial device for measuring dynamic weight-bearing ankle dorsiflexion [29] and angle positions for the assessment of joint range of motion [30]. Previous studies have also shown the satisfactory reliability and accuracy of results from the accelerometer under static and dynamic conditions [31], and proved the device to be valid and reliable for step counts, regardless of the level of physical activity [32].

The device integrates different sensors: four 3D accelerometers operating at different scales (±16 G, ±16 G, ±32 G, and ±400 G); three gyroscopes (two at ± 2000°/s at 1000 Hz and one at ± 4000°/s at 1000 Hz); a 3D magnet (±8 Gauss at 160 Hz); and a barometer (±1200 mbar at 100 Hz). For registration of the spatial positioning, speed, and acceleration, the device integrates two sensors: a global navigation satellite system and a global position system (GNSS/GPS) at 10 Hz (number of satellites = 8.96; SD = 1.56). It has four communication interfaces: WIFI 802.11 b/g/n, wireless Bluetooth, wireless ANT+, and USB 2.0 (high-speed). The recorded data are stored in a 16 GB internal flash memory. The device has an internal battery with a four-hour duration. It weighs 70 g, and its dimensions are 81 × 45 × 16 mm.

For collecting, processing, and reporting of GPS data, the guidelines of Malones et al. [33] were considered (e.g., soccer players should wear the devices in appropriate tight-fitting garments in the upper part of the back to hold the device and minimize unwanted movement; they should also ensure that devices have satellite connection (known as GPS lock) before any data collection by placing them in a clear outdoor space for a sufficient period of time to achieve GPS lock). Prior to their placement, the devices were calibrated and synchronized. Due to this process, the accelerometers eliminate the four sources of error that may occur: displacement error, scaling error, orthogonal errors, and random error [34].

The device calibration process was carried out following the manufacturer’s recommendations in the auto-start process. To ensure proper functioning, three criteria had to be met: (a) leaving the device immobile for 30 s, (b) on a flat surface, and (c) without close contact with magnetic devices [35]. Following this procedure, the accelerometers in the device obtained very high reliability values in static and dynamic tests in different anatomical locations [36].

### 2.4. Training Load

The evaluated outcomes were: (i) distance (total distance covered, meters); (ii) speed running (from 0–6 to 24–50 km·h^−1^, meters); (iii) *P*_met_ (W/kg), the product of speed and energy cost of the activity derived from inclination and acceleration [22], calculated using the following equation: PM = EC × S High; (iv) high metabolic load distance (HMLD, meters), distance traveled when *P*_met_ is > 25.5 (W/kg) [29]; and (v) Edwards’ training load (Edwards’ TRIMP), which determines the internal load by measuring a product of the accumulated training duration (minutes) of 5 HR zones by a coefficient related to each zone (50% to 60% of HRmax × 1; 60% to 70% of HRmax × 2; 70% to 80% of HRmax × 3; 80% to 90% of HRmax × 4; and 90% to 100% of HRmax × 5), expressed as a single value in arbitrary units (a.u.) [37].

### 2.5. Small-Sided Game Conditions

**Figure 1:**
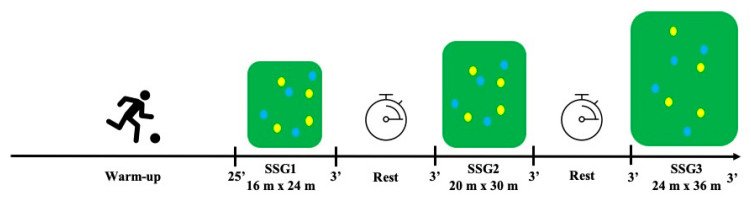
Schematic representation of the experimental protocol.

Players were randomly assigned to the playing teams, with no specific tactical missions. Since communication is a relevant element in the coach–athlete relationship [38], coaches provided feedback aiming to encourage players during all the SSGs. Soccer players, colleagues, and coaches were around the pitch with soccer balls in their hands to replace the ball each time it left the pitch (ball possession quickly changed) during all the SSGs, with 3 min duration and 3 min rest intervals. The objective of the game was for the team to keep the ball in their possession for as long as possible, starting with the pitch size of smaller dimensions in bout 1 (SSG1). Figure 1 presents the performed SSGs and their different conditions.

### 2.6. Statistical Methods

Variables were characterized using descriptive analysis (mean and standard deviation, M and SD). The normality of the distributions was assessed with the Shapiro–Wilk test. Parametric and nonparametric statistics were selected accordingly. Comparisons between age categories (U-12, U-15, and U-23) were evaluated using standardized differences with combined variance, derived from the M and SD of each variable, with 95% confidence intervals.

The Manova test was performed to compare the performance in the different SSGs. Bonferroni post-hoc test verified which pairs of means were significantly different (*p* < 0.05). Effect size (ES) was determined by calculating partial eta-squared ($\eta_{p}^{2}$) [39], considered as small ($\eta_{\rho }^{2}$ < 0.06), moderate (0.06 < $\eta_{\rho }^{2}$ < 0.15), or large ($\eta_{\rho }^{2}$ > 0.15) [40]. The data analysis was performed using the Statistical Package for Social Sciences (SPSS 25.0, SPSS Inc., Chicago, IL, USA).

## 3. Results

Table 1 presents a comparison, according to age categories, of the studied variables throughout the played SSGs. Total distance concomitantly increased with the pitch size increase in all the studied age categories. Nevertheless, significant differences were only observed across all age categories in the comparison of SSG1 (16 × 24 m) and SSG3 (24 × 36 m). Distance covered at running speed 0–6 km·h^−1^ presented a downward trend in all age categories throughout the SSGs, except in the U-12 age category transitioning from SSG2 (20 × 30 m; 106.55 ± 16.60 m) to SSG3 (117.87 ± 39.65 m). Moreover, significant differences when evaluating covered distance at 0–6 km·h^−1^ and 6–12 km·h^−1^ were only observed in U-15 and, with a different trend, the distance covered at 0–6 km·h^−1^ in SSG1 was higher compared with the other performed SSGs. At 6–12 km·h^−1^ the distance covered in SSG1 was lower compared with SSG2 and SSG3.

**Figure 2:**
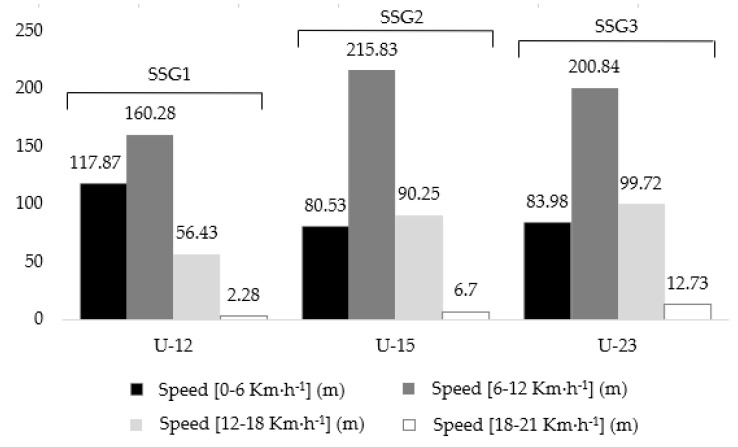
Distance covered in different running speed intervals throughout the performed SSGs by U-12, U-15, and U-15 soccer players. SSG1: 4 vs. 4, 16 × 24 m; SSG2: 4 vs. 4, 20 × 30 m; SSG3: 4 vs. 4, 24 × 36 m. All SSGs with 3 min duration and 3 min rest (ratio 1:1).

Distance covered at 12–18 km·h^−1^ increased throughout all the performed SSGs in all age categories, but significant differences were only visible in U-12 (SSG1 < SSG2 < SSG3) and U-23 (only when comparing SSG1 and SSG3). At 18–21 km·h^−1^ running speed, no distance covered was observed in U-12 in SSG1 and SSG2, but the recorded mean distance in SSG3, despite showing low values (2.28 ± 2.55 m), was different compared with previous performed SSGs in this age category. In all the other age categories, smaller values of distance covered were observed when compared with slower running speed intervals displayed in the performed SSGs (0–6, 6–12, and 12–18 km·h^−1^), but significant differences were only evident in U-23 when comparing SSG3 (12.73 ± 11.95 m) to SSG2 (12.73 ± 11.95 m). The distance covered in running speeds between 0 and 21 km·h^−1^ in all age categories and in the different SSGs in displayed in Figure 2.

Distances covered at running speeds of 21–24 and 24–50 km·h^−1^ were only observed during SSG3. In U-15 (21–24 km·h^−1^; 0.89 ± 1.80 m) and U-23 (21–24 km·h^−1^; 7.15 ± 8.85 m/24–50 km·h^−1^; 1.50 ± 1.94 m), there were significant differences for distance ran at 21–24 km·h^−1^ when comparing SSG3 with the two previously performed SSGs.

*P*_met_, HMLD, and Edwards’ TRIMP tendentially increased with age when comparing the performed SSGs. The only exceptions were observed in *P*_met_ in U-12 when considering the transition between 16 × 24 m (SSG1) to 20 × 30 m (SSG2), and in Edwards’ TRIMP in U-15 and U-23 when considering the transition from SSG2 to SSG3. Despite these observations, significant differences were only observed in U-12 when considering *P*_met_ and the transition from SSG1 to SSG3 (844.01 ± 46.34 < 930.85 ± 51.79) and HMLD, where mean values in SSG2 were significantly higher compared with SSG1 and lower compared with SSG3 (26.11 ± 6.20 < 38.65 ± 7.86 < 52.67 ± 15.10). The differences between age categories for the different SSGs, considering all the studied variables, are shown in Table 2.

Total distance, HMLD, and distance covered at running speeds in the intervals 6–12 km·h^−1^ and 12–18 km·h^−1^ were always different between age categories in all the three performed SSGs. At 0–6 km·h^−1^, no significant differences were observed in the U-23 age category in SSG2 and SSG3, unlike the other age category cohorts in these SSGs. No differences were observed regarding 18–21 km·h^−1^ in any age category and SSG. Differences in *P*_met_ were only observed in SSG2 and SSG3, considering all age categories. Distance covered at running speeds in the intervals 21–24 km·h^−1^ and 24–50 km·h^−1^ was only different in U-12 and U-23, specifically in SSG3 (24 × 36 m). With respect to Edwards’ TRIMP, differences only occurred in SSG2 (20 × 30 m) for the younger soccer players age categories (U-12 and U-15).

## 4. Discussion

The aim of this study was to evaluate the training load in different age category soccer players associated with distinct pitch size SSGs. To the best of our knowledge, this is the first study which comprehensively analyzes the total distance covered, different interval speeds, covered distance, and metabolic demand of different age category soccer players in three distinct pitch size SSGs (SSG1, 16 × 24 m; SSG2, 20 × 30 m; and SSG3, 24 × 36 m). The main results were: (i) total covered distance increased in all age categories in line with the increase in pitch size from SSG1 to SSG3, with significant differences between all age categories; (ii) distance covered at running speeds of 18–21 km·h^−1^ or above were residual or even null, particularly in the U-12 and U-15 age categories; (iii) the increase in pitch size from 16 × 24 m (SSG1) to 20 × 30 m (SSG2) and 24 × 36 m (SSG3) promoted different responses in distance covered at different running speeds when considering the different age categories of the soccer players and; (iv) *P*_met_, HMLD, and Edwards’ TRIMP increased with age in the same performed SSG and tendentially increased in the same age category with the increase in pitch size between SSG1 and SSG3.

It was previously observed that playing area dimensions influence the intensity of the game, the actions of the players, and the energy systems [13]. Large playing areas are associated with an increase in the intensity of exercise [41], while small playing areas appear to foster technical development [42]. In the present research, total distance always increased with the increase in pitch size but was never different in any of the studied age categories when considering the transition from SSG1 to SSG2 (16 × 24 m to 20 × 30 m). Significant differences were only observed when considering the comparison of total distance covered in SSG1 and SSG3 (16 × 24 m and 24 × 36 m), in all age categories.

Our results suggest that SSGs on pitch sizes from 16 × 24 m to 20 × 30 m are not the best task context for significant stimulus of total running distance in all the studied age categories, evidence reinforced through the analysis of *P*_met_ in the U-12 age category, which decreased from SSG1 to SSG2. This fact should be associated with the evidence that higher physiological strain, typically observed in SSGs performed on larger pitches, is likely to be due to the possibility of making longer offensive and defensive runs [43] in ball possession SSGs, both during ball possession and without the ball when aiming to receive a pass from a teammate.

In overall terms, SSGs typically present lower demands relating to HSR and sprint actions [44]. Nevertheless, it was recently stressed that sprint and HSR should form an integral part of the player monitoring process [19]. It was previously observed that larger playing areas and having more players per team may encourage players to increase their sprinting distance to create longer pass lines or to exploit the length of the field [45]. HSR was associated with distance covered above 16 km·h^−1^, which is supposedly the average maximal aerobic speed in professional soccer players, and sprinting, distance covered above 21 km·h^−1^, which is supposedly 60%–70% of maximal sprinting speed [22]. Additionally, a study by Gaudino et al. [21] shows that distances covered above 14.4 km·h^−1^ were greater when the area per player increased.

In our study, considering U-15 and U-23 age categories, the distance covered at 0–6 km·h^−1^ speed tendentially decreased between SSG1 and SSG3 (the only exception being in U-12, considering the transition between SSG2 and SSG3), but it should be noted that only in SSG1 and U-15 age category was it significantly higher compared with SSG2 and SSG3. Contrarily, in U-12 and U-23 for the 6–12 km·h^−1^ running speed, the distance covered always increased with the increase in pitch size, despite no significant differences being observed. Only in the U-15 age category were SSG3 and SSG2 distance covered at this running speed significantly higher compared with SSG1. Interestingly, even though distance covered at 12–18 km·h^−1^ always increased in the different studied age categories throughout the played SSGs (lower distances compared with both 0–6 and 6–12 km·h^−1^), significant differences were only observed in U-12 and U-23 players.

These results highlight that the methodological framework applied in this study does not provide the same stimuli for U-12, U15, and U-23 soccer players, since significant differences were only observed in the U-15 age category for both the 0–6 and 6–12 km·h^−1^ interval speed running distances, in contrast to what was observed in the 12–18 km·h^−1^ interval speed, where significant differences only occurred in U-12 and U-15. Another interesting fact was that the distance covered at 12–18 km·h^−1^ was significantly lower in U-12 compared with U-15, and in this age category also significantly lower compared with U-23. Associated with these facts, in all age categories and SSGs the distance covered at 12–18 km·h^−1^ was always more than 50% lower compared with that observed in 6–12 km·h^−1^. These observations from our study are particularly relevant when soccer coaches prepare SSG training tasks aiming for distance covered and particularly HSR, associated to the 12–18 km·h^−1^ running speed interval.

It was previously indicated that players require sufficient pitch dimensions and acceleration time to reach high-speed running (>19.8 km·h^−1^) and/or sprinting (>25.2 km·h^−1^) speed thresholds [44]. In our study, U-12 presented very low (SSG3, 2.28 ± 2.55 m) or even null values (SSG1 and SSG2) when considering distance covered at 18–21 km·h^−1^ running speed, with SSG3 being significantly higher compared with the previously played SSGs. In U-15 and U-23, the distance covered in this running speed interval was always small (<10 m), except in U-23 and SSG3 (12.73 ± 11.95 m), which was significantly higher compared with SSG2.

Considering the 21–24 km·h^−1^ running speed interval, values were only observed in SSG3 in U-15 (0.89 ± 1.80 m) and U-23 (7.15 ± 8.85 m), the last being significantly higher compared with SSG1 and SSG2. Running speeds of 24–50 km·h^−1^ were only observed in U-23 and during SSG3 (1.50 ± 1.94 m). Our results indicate that SSGs with pitch sizes below 24 × 36 m are predominantly performed at speeds below 18 km·h^−1^ in the studied age categories, which is the running speed that is associated with the running intensities that prevail during soccer games, and are below HSR, which is determinant of the more dynamic actions of soccer players that, in many circumstances, decide the result of the game in soccer.

*P*_met_ was previously criticized in research due to the underestimation of energy expenditure [24]; nevertheless, it was previously observed that *P*_met_ was greater when the pitch area increased [21]. In another perspective, Goto and King [23] indicated that the speed approach underestimates the high intensity demands of match play compared with the *P*_met_ approach, especially in players that cover less distance at high speeds. These authors added that coaches and sports scientists should pay attention to the methodology for monitoring players (speed vs. *P*_met_) and are advised to carefully choose the pitch size of SSGs, together with the number of players per team, depending on the aim of training sessions.

Our results indicated that *P*_met_ always increased with age in the same SSG when comparing the age categories from younger to older; however, only in U-12 was the *P*_met_ in SSG3 significantly higher compared with SSG1, and it even decreased in the transition from SSG1 to SSG2. These facts, and considering that differences between age categories only occurred in SSG2 and SGG3, suggest that the change from 16 × 24 m to 20 × 30 m pitch sizes in SGGs did not induce significant stimuli in this SSGs methodological framework for U-12, U-15, and U-23 soccer players.

The HMLD mean values presented the same trend as *P*_met_, considering both the increase in the same age category with the increase in pitch size (SSG1 > SSG2 > SSG3) was also always higher between age categories for the same performed SSG and in line with the increase in pitch size, and was different between all the studied age categories in the same performed SSG. Furthermore, significant differences only occurred in U-12 players throughout the played SSGs. Previous investigation showed that the energy expenditure and distance covered at different *P*_met_ better inform of the true physical demands imposed on players [22].

Our results reveal that training load is different considering age categories, supporting the notion that metabolic assessment is complementarily relevant when analyzing soccer SSGs performed by different age categories. The Edwards’ TRIMP analysis supported these assumptions, since differences were only observed in SSG2 between U-12 and U-15 age categories, despite no significant differences being observed in the same age category considering the rise in pitch size from SSG1 to SSG3. In this regard, it is also of note that Edwards’ TRIMP values tendentially increased in the same age category with pitch size increase, except in U-15 and U-23 and the transition from SSG2 to SSG3.

Some limitations of the present study should be considered when interpreting the findings. Firstly, the maturation of the players was not evaluated. Secondly, participants were national- and international-level male soccer players and played in teams with a specific playing system. It is unclear whether these findings can be extrapolated to other teams/players. Results should be interpreted with caution since the players’ performance in bouts 2 and 3 may have been influenced by fatigue accumulation from previous SSGs, as the purpose of the study was to evaluate the training task performed by different age category soccer players under different SSG conditions in the same training session. In the future, we suggest that the study protocol could be verified in more than one training session, in SSGs with the same pitch size, or even starting with the SSG with the larger pitch size and ending with the smaller pitch size. Likewise, other SSG constraints—for example, the inclusion of goals and goalkeepers, other pitch sizes, and the time of play and rest intervals—should also be considered in in these and other age categories, and in women’s soccer.

## 5. Conclusions

In U-12, U-15, and U-23 soccer players, distance covered increases concomitantly with the increase in pitch size in SSGs between 16 × 24 m and 24 × 36 m. The studied play and rest interval time associated with the dimensions of SSG1 (16 × 24 m) did not provide high levels of internal and external training load, nor did it result in many differences when considering the evaluated age categories (U-12, U-15, and U-23). Consequently, the use of this pitch dimension in SSGs should be considered by coaches if the focus of the training task is related to internal and external load. The design of SSGs must consider that the training load of the players differs according to their age category. Accordingly, children and/or adolescents should experience training tasks that are appropriate for their age category.

Pitch sizes below 24 × 36 m do not offer the ideal environment for HSR; more specifically, in U-12 and U-15 soccer players, specific intervention strategies or complementary exercises should be considered if the aim of the training task is HSR. Nevertheless, when considering the relationship between different running speed intervals and internal load (namely *P*_met_, HMLD, and Edwards’ TRIMP) it is possible to observe different internal and external training load levels and trends according to age categories and performed SSGs, a fact that should be considered when prescribing training tasks to different age category soccer players and which highlights the importance of considering metabolic assessment in soccer training.

Wearable technology used for the purpose of monitoring soccer players during training and competition represents a fundamental support for players performance evaluation and enhancement, also representing an important tool for coaches daily training prescription.

## Figures and Tables

**Table 1:** Comparison between small-sided soccer games according to age categories.

		SSG1	SSG2	SSG3	Manova (SSG)				
Variables	Age Category	*M ± SD*	*M ± SD*	*M ± SD*	*F*	ρ	$\eta_{p}^{2}$	π	*Post-Hoc*
Distance (m)	U-12	277.55 ± 41.85 *	299.35 ± 25.52	325.51 ± 14.49 *	5.294	0.001	0.335	0.778	1 < 3
	U-15	356.91 ± 30.41 *	383.48 ± 23.37	394.00 ± 24.97 *	4.226	0.029	0.287	0.676	1 < 3
	U-23	359.85 ± 18.87 *	374.78 ± 26.45	405.97 ± 37.30 *	4.752	0.022	0.346	9.505	1 < 3
Speed (0–6 km·h^−1^) (m)	U-12	117.00 ± 10.14	106.55 ± 16.60	117.87 ± 39.65	0.488	0.624	0.044	0.119	-
	U-15	99.14 ± 13.37 *^#^	81.61 ± 7.02 *	80.53 ± 12.54 ^#^	6.793	0.005	0.393	0.875	1 > 2; 1 > 3
	U-23	95.20 ± 15.17	91.99 ± 11.19	83.98 ± 17.15	1.079	0.361	0.107	0.209	
Speed (6–12 km·h^−1^) (m)	U-12	156.90 ± 21.80	160.26 ± 32.95	160.28 ± 21.39	0.045	0.956	0.004	0.056	-
	U-15	187.75 ± 17.68 *^#^	227.70 ± 11.85 *	215.83 ± 25.82 ^#^	9.081	0.001	0.464	0.953	1 < 2; 1 < 3
	U-23	196.91 ± 29.23	198.06 ± 21.74	200.84 ± 33.70	0.035	0.966	0.004	0.054	
Speed (12–18 km·h^−1^) (m)	U-12	16.14 ± 8.94 *^#^	32.51 ± 11.36 *^†^	56.43 ± 12.03 ^#^	27.847	0.000	0.726	1.000	1 < 2; 1 < 3; 2 < 3
	U-15	65.46 ± 25.90	72.03 ± 24.55	90.25 ± 20.86	2.315	0.123	0.181	0.417	-
	U-23	65.34 ± 15.57 *	82.40 ± 28.24	99.72 ± 26.20 *	3.593	0.049	0.285	0.589	1 < 3
Speed (18–21 km·h^−1^) (m)	U-12	0 ± 0 *	0 ± 0 ^#^	2.28 ± 2.55 *^#^	6.408	0.007	0.379	0.855	1 < 3; 2 < 3
	U-15	1.78 ± 5.04	1.95 ± 2.61	6.70 ± 6.22	2.632	0.095	0.200	0.466	-
	U-23	2.37 ± 2.53	2.30 ± 3.56 *	12.73 ± 11.95 *	13.105	0.002	0.421	0.928	2 < 3
Speed (21–24 km·h^−1^) (m)	U-12	0 ± 0	0 ± 0	0 ± 0	-	-	-	-	-
	U-15	0 ± 0	0 ± 0	0.89 ± 1.80	1.965	0.165	0.158	0.360	-
	U-23	0 ± 0 *	0 ± 0 ^#^	7.15 ± 8.85 *^#^	4.569	0.047	0.202	0.525	1 < 3; 2 < 3
Speed (24–50 km·h^−1^) (m)	U-12	0 ± 0	0 ± 0	0 ± 0	-	-	-	-	-
	U-15	0 ± 0	0 ± 0	0 ± 0	-	-	-	-	-
	U-23	0 ± 0	0 ± 0	1.50 ± 1.94	4.208	0.055	0.189	0.493	-
Metabolic Power (W/kg)	U-12	844.01 ± 46.34 *	815.18 ± 68.32	930.85 ± 51.79 *	4.956	0.017	0.321	0.719	1 < 3
	U-15	917.80 ± 336.04	1112.87 ± 75.65	1151.38 ± 74.15	3.032	0.070	0.224	0.525	-
	U-23	1097.89 ± 77.50	1132.35 ± 96.35	1188.72 ± 137.84	1.288	0.300	0.125	0.243	-
High Metabolic Load Distance (>25.5 W/kg) (m)	U-12	26.11 ± 6.20 *^#^	38.65 ± 7.86 *^†^	52.67 ± 15.10 ^#†^	12.902	0.000	0.551	0.992	1 < 2; 1 < 3; 2 < 3
	U-15	62.85 ± 15.74	64.13 ± 14.95	76.47 ± 15.15	1.937	0.169	0.156	0.356	-
	U-23	73.57 ± 14.55	78.09 ± 24.09	94.92 ± 25.20	1.862	0.184	0.171	0.336	-
Edwards’ TRIMP (a.u.)	U-12	10.90 ± 1.69	11.20 ± 1.69	12.11 ± 1.49	1.177	0.328	0.101	0.230	-
	U-15	11.97 ± 1.66	12.45 ± 1.56	12.33 ± 1.45	0.204	0.817	0.019	0.078	-
	U-23	12.38 ± 1.52	13.96 ± 0.56	13.42 ± 1.11	3.470	0.053	0.278	0.574	-

SSG1: 4 vs. 4, 16 × 24 m; SSG2: 4 vs. 4, 20 × 30 m; SSG3: 4 vs. 4, 24 × 36 m. All SSGs with 3 min duration and 3 min rest (ratio 1:1). *,^#^, and ^†^ represent significant differences.

**Table 2:** Differences between age categories in different small-sided soccer games.

SSGs	Variables	*F*	ρ	$\eta_{p}^{2}$	π	*Post-Hoc*
1	Distance (m)	16.303	0.000	0.620	0.998	U12 < U15; U12 < U23
	Speed (0–6 km·h^−1^) (m)	6.205	0.008	0.383	0.840	U12 > U15; U12 > U23
	Speed (6–12 km·h^−1^) (m)	6.340	0.007	0.388	0.848	U12 < U15; U12 < U23
	Speed (12–18 km·h^−1^) (m)	18.861	0.000	0.654	1.000	U12 < U15; U12 < U23
	High Metabolic Load Distance (>25.5 W/kg)	29.066	0.000	0.744	1.000	U12 < U15; U12 < U23
2	Distance (m)	26.813	0.000	0.728	1.000	U12 < U15; U12 < U23
	Speed (0–6 km·h^−1^) (m)	8.295	0.002	0.453	0.931	U-12 > U-15
	Speed (6–12 km·h^−1^) (m)	16.075	0.000	0.616	0.998	U12 < U15; U12 < U23
	Speed (12–18 km·h^−1^) (m)	10.764	0.001	0.518	0.977	U12 < U15; U12 < U23
	Metabolic Power (>25.5 W/kg)	25.930	0.000	0.718	1.000	U12 < U15; U12 < U23
	High Metabolic Load Distance (>25.5 W/kg)	11.066	0.001	0.525	0.980	U12 < U15; U12 < U23
	Edwards’ TRIMP (a.u.)	7.223	0.004	0.419	0.893	U-12 < U15
3	Distance (m)	20.608	0.000	0.673	1.000	U12 < U15; U12 < U23
	Speed (0–6 km·h^−1^) (m)	4.832	0.019	0.326	0.734	U12 > U15
	Speed (6–12 km·h^−1^) (m)	8.948	0.002	0.472	0.948	U12 < U15; U12 < U23
	Speed (12–18 km·h^−1^) (m)	9.733	0.001	0.493	0.963	U12 < U15; U12 < U23
	Speed (21–24 km·h^−1^) (m)	4.504	0.024	0.311	0.702	U12 < U23
	Speed (24–50 km·h^−1^) (m)	4.879	0.019	0.328	0.738	U12 < U23
	Metabolic Power (>25.5 W/kg)	17.552	0.000	0.637	0.999	U12 < U15; U12 < U23
	High Metabolic Load Distance (>25.5 W/kg)	9.623	0.001	0.490	0.962	U12 < U15; U12 < U23

SSG1: 4 vs. 4, 16 × 24 m; SSG2: 4 vs. 4, 20 × 30 m; SSG3: 4 vs. 4, 24 × 36 m. All SSGs with 3 min duration and 3 min rest (ratio 1:1).

## Data Availability

The data that support the findings of this study are available from the corresponding authors (mario.espada@ese.ips.pt and fernando.santos@ese.ips.pt), upon reasonable request.
